# Diversity of Caregiving Experiences in the United States: Findings From the National UAS-CLEAR Study

**DOI:** 10.1093/geroni/igaf122.2081

**Published:** 2025-12-31

**Authors:** Kira Birditt, Marco Angrisani, Angela Turkelson, Angelica Eagle, Crystal Ng, Joan Monin

**Affiliations:** University of Michigan, Ann Arbor, Michigan, United States; University of Southern California, Los Angeles, California, United States; University of Michigan Institute for Social Research, Ann Arbor, Michigan, United States; University of Michigan, Ann Arbor, Michigan, United States; University of Michigan, Ann Arbor, Michigan, United States; Yale, Yale, Connecticut, United States

## Abstract

Given the rising number of older adults with disability and dementia, unpaid caregivers are increasingly vital in the long-term care of older adults. Caregiving is often provided by multiple types of social ties (e.g., family, and non-family); yet, most research focuses on spouse and adult child caregivers and lacks information regarding caregivers across the adult lifespan who may have unique caregiving experiences. The purpose of this study is twofold: 1) describe the frequency of caregivers by relationship type (care for spouse, parent, other family, non family), dementia status, and age of caregiver, and 2) examine whether there are differences in their reports of caregiving intensity (i.e., number of caregiving tasks), caregiver burden, and rewards. We used data from UAS-CLEAR; a national study of caregivers in the U.S. (N = 2202; 67% women; 56% white; aged 18 to 102; M = 50). Caregivers were most likely to care for other family, followed by parent, spouse, and non-family. A total of 22% were caring for an individual with dementia. Non-family caregivers reported the least burden and lower caregiving intensity, whereas spouse caregivers reported the greatest burden and caregiving intensity. Older caregivers reported less burden and less caregiving intensity than younger caregivers. Individuals who cared for individuals with dementia reported greater burden and caregiving intensity, and fewer rewards than those caregiving for individuals without dementia. In sum, caregiving is not universally burdensome and younger spousal caregivers for those living with dementia may be at greatest risk.

